# Water Mediation Is Essential to Nucleation of β-Turn Formation in Peptide Folding Motifs**

**DOI:** 10.1002/anie.201307657

**Published:** 2013-10-15

**Authors:** Sebastian Busch, Chrystal D Bruce, Christina Redfield, Christian D Lorenz, Sylvia E McLain

**Affiliations:** Department of Biochemistry, University of OxfordSouth Parks Road, Oxford, OX1 3QU (United Kingdom); Department of Chemistry, John Carroll University University HeightsOH 44118 (USA); Department of Physics, King's College LondonLondon WC2R 2LS (United Kingdom)

**Keywords:** β-turns, hydrogen bonds, peptides, structure elucidation, water

Protein folding is evidently not a random process given the speed and reproducibility of folding in vivo;^1^ yet how a given polypeptide sequence translates into the globular structure of a fully folded protein remains unclear,^2^ particularly with respect to the role that water plays in this process.^3^ One frequently occurring folding pattern in proteins is the β-turn,^4^ where the amino acid sequences that give rise to these turns are thought to nucleate folding.^5^ The question remains however, if it is the mere presence of certain amino acids which initiate the formation of β-turns or if water plays a fundamental role in this process.

The hydrophilic/hydrophobic nature of peptides and proteins in physiological solutions can be probed using neutron diffraction enhanced by isotopic substitution (NDIS). NDIS can directly address structural interactions between water and biomolecules in solution^6^–^8^—the physical milieu in which these life-giving molecules must operate.

The glycine-proline-glycine sequence in the peptide GPG-NH_2_ is known to occur in β-turns in proteins.^9^, ^10^ Its structure in aqueous solution (Figure 1) has been assessed using NDIS in concert with NMR spectroscopy and both molecular dynamics (MD) and empirical potential structural refinement (EPSR) simulations. This unique combination of techniques allows for structural interactions between GPG-NH_2_ and water to be investigated on the atomic scale (10^−10^ m, Å), the scale of hydrogen-bonding interactions; yielding a full assessment of the role that water plays in peptide conformation in solution and, importantly, how this relates to peptide folding.

**Figure 1:**
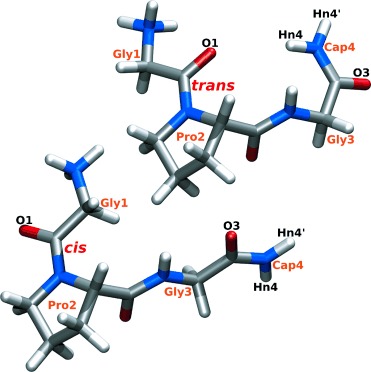
Molecular structure of *trans* and *cis* GPG-NH_2_ molecules.

Radial distribution functions (*g*(*r*)s)—which show the average distances in solution—for water atoms (Hw/Ow) around the Gly1-Pro2 peptide bond oxygen (O1) and the Gly3-Cap4 peptide bond oxygen (O3) from EPSR and MD are shown in Figure 2 a. The EPSR simulation contained a mixture of *cis* and *trans* GPG-NH_2_ molecules in a ratio corresponding to that measured by ^1^H NMR spectroscopy and the MD *g*(*r*) functions are from two simulations—one which contained only *cis* peptides and one containing only *trans* peptides.

**Figure 2:**
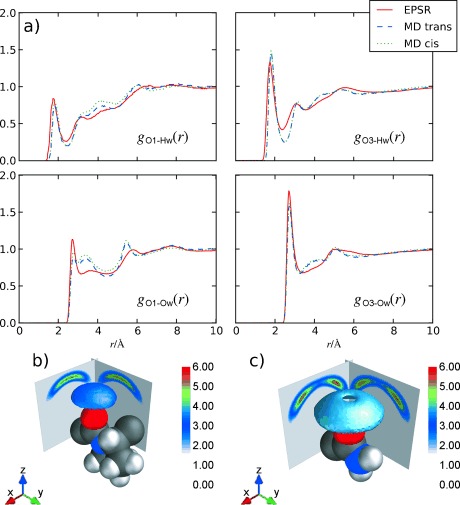
a) Radial distribution function (*g*(*r*)) for water (Hw/Ow) peptide bond oxygen atoms O1 and O3 of GPG-NH2 from MD *cis* versus *trans* and EPSR. b) Spatial density function (SDFs) of Ow around O1 and around O3 (c) from EPSR. The 3D shells show the top 30 % of water molecules from 2.0 to 3.0 Å from the origin (the center of O1 or O3). Cuts through the 3D distributions in the *xz* and *yz* planes are also shown, offset by 3.0 Å for clarity. The proline ring (O1; b) is shown in a *trans* conformation but the EPSR simulation contains a mixture of both conformers.

The reduction of intensity in the first peak of the *g*_O1-Hw_(*r*) compared to the *g*_O3-Hw_(*r*) and the average coordination from these peaks shows fewer Hw-O1 hydrogen bonds (1.1) than O3-Hw bonds (1.7). This indicates that the Gly1-Pro2 peptide bond oxygen is not fully hydrated with respect to both the Gly3-Cap4 peptide bond oxygen and to previous measurements of C=O hydration.^8^

Spatial density functions,^11^ which show the most probable location of water molecules around O1 and O3 are shown in Figure 2b and c. Here, water molecules around O1 are preferentially located directly above the C=O group, in a fairly tight distribution, indicating highly directed hydrogen bonding from water to this oxygen. In contrast, O3 shows a much broader distribution, similar to that seen for acetylcholine in aqueous solution where the C=O group in this neurotransmitter is highly accessible to the bulk water solvent.^7^

That there are more highly directed water molecules around O1 is further evident on comparison of the *g*_O1-Ow_(*r*) and *g*_O3-Ow_(*r*) functions in Figure 2 a; the *g*_O1-Ow_(*r*) shows a more shallow minimum after the first peak compared to the *g*_O3-Ow_(*r*). This shallow minimum in the O1-Ow function gives rise to a small peak at about 3.5 Å in the MD simulations and a smaller peak at the same distance in the EPSR simulations, while O3-Ow shows the more usual effect of water around fully hydrated oxygen, with a single sharp peak at about 3 Å. Interestingly, the O1-Ow hydration in the MD simulations look similar when the molecule is *cis* or *trans* suggesting that the proline ring has a steric influence on the O1 hydration shell, regardless of the Gly1-Pro2 peptide bond conformation.

The ^1^H NMR spectrum of GPG-NH_2_ gives an 85:15 mixture of *trans*:*cis* conformations about the Gly1-Pro2 peptide bond, as expected for cationic proline-containing peptides in solution.^12^ Interestingly, the terminal hydrogen atoms on the -NH_2_ of GPG-NH_2_, Hn4, and Hn4′ shown in Figure 3 a, also show distinct peaks corresponding to *cis* and *trans* conformers, even though this group is separated from the Gly1-Pro2 peptide bond by seven bonds.

**Figure 3:**
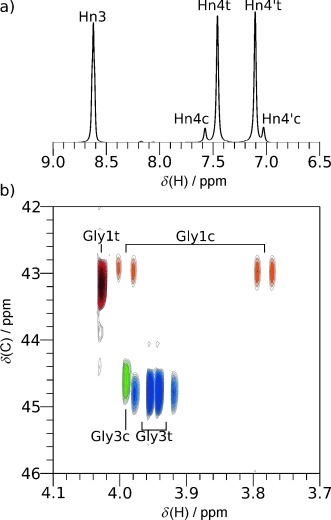
a) Downfield region of the ^1^H NMR spectrum for GPG-NH_2_ in H_2_O; peaks from the *cis* (c) and *trans* (t) conformers are shown. b) A portion of the ^1^H–^13^C HSQC NMR spectrum for GPG-NH_2_ in D_2_O showing CH_2_ groups of Gly1 and Gly3. Gly1 shows nonequivalent H_α_ when GPG-NH_2_ is in a *cis* conformation and Gly3 shows this when GPG-NH_2_ adopts the *trans* conformation. The full ^1^H and HSQC spectra are shown in the Supporting Information.

The region of the ^1^H–^13^C HSQC spectrum for GPG-NH_2_ glycine H_α_–C_α_ correlations is shown in Figure 3 b. The peaks at about 43 ppm in the ^13^C dimension are from Gly1 and the peaks at about 45 ppm from Gly3. For Gly1, the single peak at 4.15 ppm in the ^1^H dimension is from the *trans* conformer; the two H_α_ are equivalent, indicating significant conformational averaging likely arising from nearly free rotation about the C_α_-C=O bond (*ψ* angle) when GPG-NH_2_ is *trans*. In the *cis* conformer, two distinct H_α_ peaks are observed for Gly1 at 3.99 and 3.78 ppm in Figure 3 b indicating restricted rotation about *ψ*. The opposite pattern is observed for Gly3, a single H_α_ peak is observed for Gly3 when Gly1-Pro2 is *cis* while a pair of H_α_ peaks, at 3.97 and 3.93 ppm, is observed when Gly1-Pro2 is *trans*; indicating that the conformation of Gly3 is more constrained when this bond is *trans*.

This restricted rotation for Gly1 in *cis* GPG-NH_2_ molecules is likely the result of steric clash between the NH_3_^+^ terminus and the rest of the GPG-NH_2_ molecule. Gly3, on the other hand, experiences more restricted rotation when the Gly1-Pro2 bond is in its more dominant *trans* configuration, indicating that Gly3 shows preferred orientations in this conformation. This preferred orientation in GPG-NH_2_ must be due to an interaction with the rest of the molecule not present in the *cis* form. The observation of distinct peaks for Hn4/Hn4′ in the *cis* and *trans* conformers (Figure 3 a) is further evidence of a difference in the behavior of Gly3 in the *cis* and *trans* GPG-NH_2_ conformers in solution.

Figure 4 shows the average inter-peptide radial distribution function *g*(*r*) between O1 and the NH_2_ terminal hydrogen (Hn4) from EPSR and MD simulations. *Trans* GPG-NH_2_ shows three broad Hn4-O1 peak maxima at around 2, 4, and 6.5 Å in the MD simulations, whereas the *cis* GPG-NH_2_ molecules are fully extended in solution and show only a large broad peak at around 8 Å. The MD *trans g(r)* indicates that GPG-NH_2_ has some association between its Gly1 and Cap4 ends; EPSR shows shorter distances for these second two peaks—at about 3.1 Å and 6 Å—and no peak at the shortest distance. The peak at 2 Å in the MD indicates a direct hydrogen bond between O1 and Hn4 whereas the peak at 3–4 Å is indicative of a more highly ordered interaction between Hn4 and O1, not because of direct hydrogen bonding between C=O and N-H groups.

**Figure 4:**
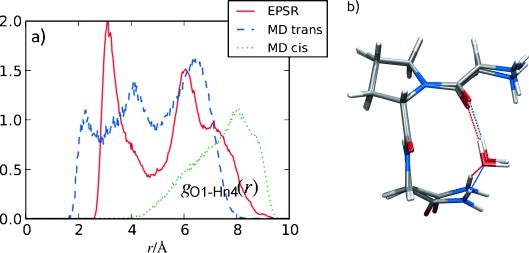
a) Intra-peptide *g*_O1-Hn4_(*r*) for EPSR and MD simulations of GPG-NH_2_ in solution; the red line is from the EPSR fits to the neutron data, the blue dashed line is the MD *trans* simulation, and the dotted green line the MD *cis* simulation. b) Representative C=O⋅⋅⋅Hw-Ow⋅⋅⋅H–N interactions with one bridging water distance from the MD *trans* (blue line) and EPSR simulations (red line).

The unique hydration structure seen in Figure 2 around O1 coupled with the distinct distances observed in Figure 4 are suggestive of a water-mediated hydrogen bonding motif between the NH_2_ and Gly1-Pro2 peptide bond oxygen. In the MD simulations, the reduction of hydration around O1 can be partially explained by the NH_2_ group directly bonding to O1, replacing some of the water molecules which would be present if the Gly1 C=O group were fully solvent accessible. However this accounts for only 3 % of the molecules and there are no direct C=O⋅⋅⋅H–N interactions apparent in the EPSR. An alternate explanation to direct intra-peptide bonding is that the more highly ordered water molecules around O1 mediate the Cap4-Gly1 interactions in solution. The O1-Ow SDF in Figure 2 b is consistent with this view, as the nearest-neighbor water molecules are preferentially oriented directly above O1 compared with the more highly solvent accessible O3 oxygen. Bridging water molecules above the Gly1 peptide oxygen would almost certainly displace hydrating water molecules, giving rise to the unique O1-Ow hydration observed in Figure 2 a.

The coordination number of the first peak (at 5 Å) in the EPSR fits to the NDIS data indicates that roughly 17 % of the molecules are likely to be mediated by one water molecule forming an O1⋅⋅⋅Hw-Ow⋅⋅⋅Hn4 interaction. The *trans* MD molecules show roughly the same coordination at a somewhat larger distance, although the exact number of molecules which are bound in this manner is difficult to assess as the *g*(*r*) functions in Figure 4 also account for GPG-NH_2_ conformations that may not contain mediating waters between O1 and Hn4.

Although at first glance the MD and EPSR intra-peptide O1-Hn4 at the distances indicative of O1⋅⋅⋅Hw-Ow⋅⋅⋅Hn4 interactions appear remarkably different at 4.0 Å and 3.1 Å, respectively, both of these distances lead to fairly similar water-mediated molecular conformations of GPG-NH_2_ as shown in Figure 4 b. In this Figure the O1-Ow and O1-Hn4 distances were set to the value of the peak maxima in Figures 2 and 4 and the Hn4-Ow distances at 1.9 Å for EPSR and 2.0 Å for MD, the value of first peak maxima in the *g*_Hn4-Ow_(*r*)s. The differences in water orientation between the single water-mediated O1⋅⋅⋅Hw-Ow⋅⋅⋅Hn4 interactions in Figure 4 b may be indicative of slightly different energetic configurations of these interactions from MD versus EPSR simulations. Interestingly, the average O1⋅⋅⋅Ow⋅⋅⋅Hn4 angle is 82° for EPSR simulations and 110° for MD simulations. By comparison the value for pure water is 86° (Ow⋅⋅⋅Ow⋅⋅⋅Hw; see the Supporting Information) when considering only hydrogen-bonding interactions between molecules. It should be noted that it is possible that the peptides themselves will adopt slightly different conformations in solution to compensate for these potentially higher-energy water-mediated configurations, thus leading to small changes in the overall energy of the system. It should also be noted that MD also shows similar configurations to EPSR in solution as there is still an appreciable amount of density in the MD intra-peptide *g*(*r*) at 3.1 Å.

Figure 5 shows the probability distribution from the *trans*-GPG-NH_2_ of directly hydrogen-bonded (C=O⋅⋅⋅H–N) molecules compared with those bonded by water-mediated interactions through one or two water molecules from the MD simulations, normalized to the total number of GPG-NH_2_ molecules in solution. To generate these distributions, only the peptides which have strongly correlated interactions bound directly to water molecules were included; O1-Hn4 distances were discounted if there were no water molecules bound through both O1-Hw and Ow-Hn1 interactions. Roughly 16 % of the molecules are either directly bound or bound by one or two water-mediated hydrogen bonds, where the average O1-Ow distance is 2.78 Å for Hn4-O1 single-water-mediated bonds, consistent with the distances observed in the *g*_O1-Ow_(*r*) in Figure 2 a.

**Figure 5:**
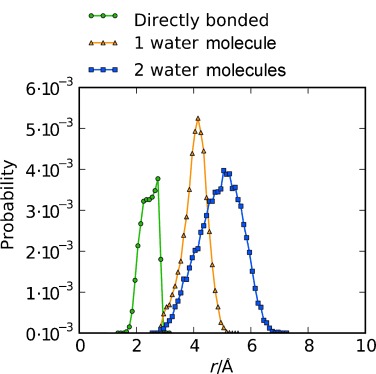
The probability of directly bonded GPG-NH_2_ molecules extracted from the *trans*-GPG-NH_2_ MD simulation for GPG-NH_2_ which has a direct bond between O1 and Hn4 atoms (green), GPG-NH_2_ molecules where O1⋅⋅⋅Hn4 is mediated by one water molecule (orange), and two water molecules (blue).

Many theories of how proteins fold are centered around the “hydrophobic effect” where the expulsion of water from hydrophobic amino acid side chains is thought to drive a structural collapse leading to a fully folded functional protein in vivo.^13^ Even though this effect is often cited as the dominant force in protein folding and association, this prevailing view has been recently challenged.^6^, ^14^ Previous work has also suggested that hydrophilic interactions could, in fact, be more important to the folding process than hydrophobic ones.^15^

It is certainly true that water should play some role as proteins fold in aqueous solutions. In this work all of the experimental and computational methods used indicate that water acts as a guide or mediator for the nucleation of folding by virtue of hydrogen-bonding interactions rather than only by a process “de-wetting” or hydrophobic elimination which has been observed in simulations of a β-hairpin-forming peptide.^16^ When GPG-NH_2_ is *trans*, a water-mediated hydrogen bond between the Gly1 O1 and Cap4 Hn4 appears to act as a nucleation point for folding before, perhaps, being finally eliminated in the fully folded protein. This indicates that water may play a dual role in β-turn formation, where perhaps water nucleates or initiates folding by mediating the formation of an *i*+4 hydrogen bond. This electrostatic water bridge would allow the hydrophobic amino acid side chains to be in close enough proximity to one another for the subsequent hydrophobic collapse to occur, leading ultimately to the formation of a functional, globular protein.

In both EPSR and MD, *trans* GPG-NH_2_ molecules are for the most part at least partially folded as the intra-peptide distances in Figure 4 suggest, as opposed to the *cis* conformers that are fully extended in solution. The contribution of water to the initiation of folding is also reflected in the unique hydration structure around the C=O (O1) oxygen (Figure 2). If this C=O oxygen was fully solvent accessible a similar hydration to the O3 C=O oxygen would be expected. The only other factor which might lead to this unique hydration would be large-scale association between separate GPG-NH_2_ molecules. However, large-scale aggregation was excluded by small-angle neutron scattering (SANS) measurements and aggregation was not evident in the simulations (see the Supporting Information). Importantly, a number of the GPG-NH_2_ intra-peptide contacts in these partially folded states appear to be mediated by water molecules which perhaps provide the impetus for nucleation mechanism of folding.

Anfinsen hypothesized that certain “portions of a protein chain that can serve as nucleation sites for folding will be those that can ”flicker“ in and out of the conformation that they occupy in the final protein.”^17^ That these peptides are “flickering” in and out of a suitable conformation is evident as the average structure in solution presented here only shows a relatively small proportion of peptides being bound by water mediation at any given time. In much the same way as GPG-NH_2_ is in solution, the bridging water molecules will likely also “flicker” in and out of the exact configuration which leads to a mediating hydrogen bond joining the C=O⋅⋅⋅H–N groups of the peptide.

That C=O⋅⋅⋅H–N bonds are necessary for the formation of a β-turn was first identified by crystallographic techniques.^18^ However, as more crystallographic data appeared in the literature for a variety of peptides and proteins which contain turn motifs,^19^ this hydrogen bonding interaction was often discounted as the distances or alignment between the C=O and H–N groups were deemed too far for hydrogen bond formation to occur.^10^, ^20^ In light of the data presented here, an alternative explanation may be that the C=O⋅⋅⋅H–N contacts are stabilized by water-mediated hydrogen bonds as a large number of β-turns in proteins are located on the protein surface,^21^ leaving them exposed to the surrounding water solvent.

For GPG-NH_2_ in water, hydrogen-bonding interactions appear to be the primary driving force in inducing this common β-turn sequence to fold. It is highly likely that hydrophilic forces are just as important in driving protein folding as the hydrophobic effect in solution, especially for the initiation of this process in vivo.

## Experimental Section

Glycyl-l-prolyl-glycinamide⋅HCl (GPG-NH_2_⋅HCl) was purchased from Bachem (Bubendorf, CH) and was used without further purification. Details of the sample preparation for NDIS and NMR are found in the Supporting Information. NMR measurements were performed on 500 and 750 MHz spectrometers (Oxford) controlled by GE/Omega software and equipped with a home-built triple-resonance pulsed-field-gradient probe head.

NDIS measurements were performed on 1 m GPG-NH_2_⋅HCl solutions using the SANDALS instruments at the ISIS Facility (STFC, UK). The EPSR^[22]^ modeling boxes contained 20 GPG-NH_3_^+^ ions, 20 Cl^−^ ions, and 1160 water molecules and the “seed” potentials were modified from the MD potentials. The peptide bonds were constrained to be planar and the *cis*/*trans* ratio was fixed to the NMR value. Both MD *cis* and *trans* simulations each contained 64 GPG-NH_3_^+^ ions, 64 Cl^−^ ions, and 3712 water molecules. GPG-NH_3_^+^ and Cl^−^ ions were modeled using the CHARMM force field and TIP3P water molecules for this force field.^23^ Water bonds and angles were constrained using the SHAKE algorithm^24^ and simulations were conducted using GROMACS.^25^ The ensemble-averaged site-site radial distribution functions (*g*(*r*)s) and the SDFs^11^ were calculated from EPSR molecular assemblies. Details of EPSR and MD simulations are shown in the Supporting Information.

## Supporting Information

As a service to our authors and readers, this journal provides supporting information supplied by the authors. Such materials are peer reviewed and may be re-organized for online delivery, but are not copy-edited or typeset. Technical support issues arising from supporting information (other than missing files) should be addressed to the authors.

miscellaneous_information
