# Supplementary material for: Water Mediation Is Essential to Nucleation of β-Turn Formation in Peptide Folding Motifs
Source: Angew Chem Int Ed Engl. 2013 Oct 15;52(49):13091–5. doi: 10.1002/anie.201307657 (PMC4227566; doi:10.1002/anie.201307657)
Supplement: Supplementary file 1 — miscellaneous_information [file anie0052-13091-SD1.pdf]

Angewandte Chemie
Eine Zeitschrift der Gesellschaft Deutscher Chemiker

Supporting Information

© Wiley-VCH 2013

69451 Weinheim, Germany

Water Mediation Is Essential to Nucleation of β-Turn Formation in Peptide Folding Motifs**

*Sebastian Busch, Chrystal D. Bruce, Christina Redfield, Christian D. Lorenz, and Sylvia E. McLain**

anie_201307657_sm_miscellaneous_information.pdf

Sample preparation

Glycyl-L-prolyl-glycinamide·HCl (GPG ·HCl) was purchased from Bachem (Bubendorf, Switzerland) and used without further purification. For the samples which required deuterium labeling, GPG ·HCl was dissolved and then lypholized in 99.8% D_2O. This process was repeated three times to ensure an adequate level of deuteration of the exchangeable hydrogens.

For the NMR experiments all samples were prepared under an inert N_2 atmosphere, either in a glove box or using a gas/vacuum manifold. D_2O (99.8%) was obtained from Acros Organics and ultrapure H_2O from a Millipore water purification system and both were thoroughly degassed using N_2 prior to use.

Deuterium-exchanged GPG (dGPG) was diluted in either D_2O and/or H_2O where two different concentrations were prepared, a 'dilute' sample at around 0.09 M and a 'concentrated' sample at the same solute:solvent ratio that was used in the NDIS, specifically at a molecular ratio of 1:58 GPG:water (0.95 M). Both dilute and concentrated samples were also prepared using fully proteated GPG and H_2O in order to measure density and pH. The pH for the dilute sample was 5.3 and 4.9 for the concentrated sample where this sample has a measured density of 1.08 g/ml in H_2O.

Neutron diffraction (NDIS) experiments and EPSR

The use of NDIS for hydrogen-containing systems is particularly effective because the neutron scattering length b of hydrogen is relatively large (-3.74 fm) and very different than that of deuterium (6.67 fm).[1] By measuring isotopically unique yet chemically equivalent species, it is possible to obtain a variety of diffraction patterns for the liquids in question. The diffraction pattern (or static structure factor) for a liquid or solution, $F(Q)$, can be written as

$$F(Q) = \sum_{\alpha,\beta\geq\alpha} (2-\delta_{\alpha\beta}) c_\alpha c_\beta b_\alpha b_\beta (S_{\alpha\beta}(Q)-1) \qquad \text{(S1)}$$

where c_i and b_i are the relative concentration and scattering length of atom i, respectively, $\delta_{\alpha\beta}$ is the Kronecker delta, Q is the scattering vector, $Q = 4\pi/\lambda \cdot \sin(2\theta/2)$ with the neutron wavelength λ and the scattering angle 2θ. Eq. S1 describes the sum of all of the partial structure factors $S_{\alpha\beta}(Q)$ for each unique atom pair. The Fourier transform of partial structure factors $S_{\alpha\beta}(Q)$ gives the atomic distances in real space, $g_{\alpha\beta}(r)$ (RDFs) on the Å (10^{-10} meters) scale *via*

$$S_{\alpha\beta}(Q) = 1 + \frac{4\pi\rho}{Q} \int r \cdot (g_{\alpha\beta}(r)-1) \cdot \sin(Qr)\, \mathrm{d}r \qquad \text{(S2)}$$

where ρ is the atomic number density of the sample (in atoms/Å^3) and $g_{\alpha\beta}(r)$ is the radial distribution function (RDF) between atoms α and β. Further, the average number of β atoms around a central α atom (the coordination number or CN) in a distance between r_1 and r_2 can be calculated from these RDFs using

$$n_\alpha^\beta = 4\pi\rho\, c_\beta \int_{r_1}^{r_2} r^2\, g_{\alpha\beta}(r)\, \mathrm{d}r \quad . \qquad \text{(S3)}$$

Neutron diffraction measurements were performed on the SANDALS instrument located at the ISIS Facility (STFC, UK) on GPG-NH_3^+·Cl^- (see Fig. 1 main text) using four isotopically substituted water solvents, each at a concentration of $\approx$ 1 M (1:58 GPG:water ratio) at 298K.

All samples for NDIS measurements were prepared by weight and then transferred to flat plate vanadium cells which were coated with an $\approx$ 0.1 mm layer of PTFE. Vanadium containers were used as the scattering of neutrons from vanadium is predominantly incoherent and thus leads to a more tractable data analysis. Although the PTFE itself contributes to the container background, the very low quantity present does not interfere with the scattering signal of the sample itself. For each measurement, the raw data obtained were converted to $F(Q)$ after appropriate corrections for absorption, multiple scattering and inelasticity effects were made using the program GUDRUN which is available at ISIS[2] based on the ATLAS package.[3] The corrected $F(Q)$ data are shown in Fig. S1 for the measured data sets listed in Table S1.

Table S1. Samples measured by neutron diffraction. hGPG denotes GPG-NH_2 ($C_9H_{17}N_4O_3^+ \ldots Cl^-$), dGPG denotes GPG-$ND_2$ ($C_9H_{11}D_6N_4O_3^+ \ldots Cl^-$).

Sample	water	h/dGPG
I	H_2O	hGPG
II	25% D_2O/75% H_2O	25% dGPG/75% hGPG
III	50% D_2O /50% H_2O	hGPG
IV	D_2O	dGPG

Empirical Potential Structure Refinement (EPSR) mod-

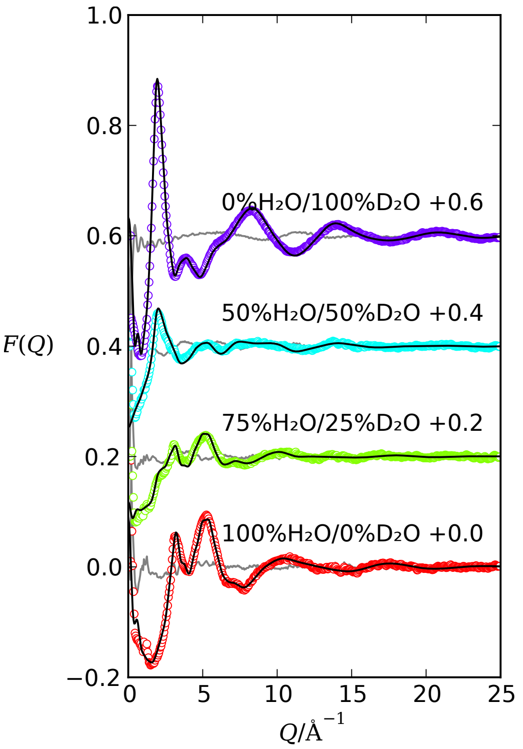

Figure S1. Data (points) and fits (black line). The deviation between data and fits are shown as grey lines.

eling is a reverse Monte Carlo modelling technique used to augment the information obtained by NDIS on solutions.[4] EPSR simulations were performed with a box of molecules that contained 20 GPG-NH_3^+ molecules, 20Cl^- ions and 1160 water molecules. The 'seed' potentials for the GPG-NH_3^+ molecules were adapted from the CHARMM force field [5,6] and modified TIP3P potentials for used for the water molecules.[7,8] The peptide bonds were constrained to be planar and the *cis-trans* ratio was fixed to the value obtained by NMR.

EPSR begins with a standard Monte Carlo simulation using a set of atomic reference potentials. After this initial step, these potentials are refined, iteratively, until the simulated structure fits all of the provided neutron data, ultimately resulting in a model which is consistent with the set of isotopically unique, chemically equivalent data. It should be noted that while EPSR provides a model which is consistent with the diffraction data, it is not necessarily unique.[4,9]

Absence of GPG-GPG aggregation in solution

Small angle neutron scattering (SANS) measurements were performed on proteated GPG in D_2O (the exchangeable hydrogens were deuterated in this instance) to assess any macromolecular structure formation in solution using the SANS2d instrument (STFC, UK) at the ISIS facility. SANS is useful in that it can detected larger range structures, typically those above around 50 Å.[10] Fig. S2 shows the SANS measurement of GPG in solution where it is evident that there are no long range structures being formed as aggregation of molecules would lead to a strong rise of the scattering intensity at low Q.

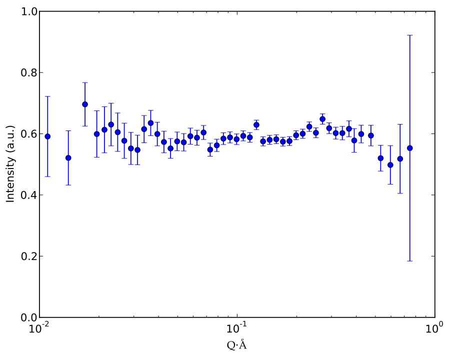

Figure S2. SANS data for GPG in D_2O.

Possible smaller scale association between GPG molecules in solution was also assessed in both EPSR and MD simulations. The *inter*molecular $g(r)$ between the GPG oxygen and hydrogen atoms on different GPG molecules are shown in Fig. S3 and the corresponding coordination numbers are listed in Tables S2, S3 and S4. Neither the pure MD simulation nor the EPSR simulation of the NDIS show an appreciable amount of aggregation.

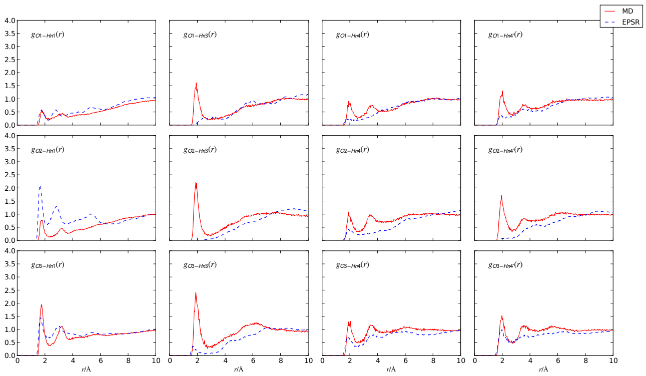

Figure S3. Intermolecular radial distribution functions of the N-H hydrogens around the C=O oxygens. The corresponding coordination numbers are given in tables S2, S3 and S4.

Details of the MD simulations

The Molecular Dynamics simulations were performed with GROMACS.[11] For each system, a series of position restraints were used first in order to eliminate any clashes between atoms that resulted from the building of the initial configurations. Then the temperature was equilibrated by using the NVT ensemble to run a 2 ns simulation at 300 K.

Table S2. Intermolecular coordination numbers (CN) at the distances given in the table as extracted from the neutron scattering data via EPSR simulations. It can be seen that the coordination numbers are small, i. e. there is no appreciable aggregation of different molecules in the simulation.

	Hn1 (2.3 Å)	Hn3 (2.8 Å)	Hn4 (2.5 Å)	Hn4′ (2.5 Å)
O1	0.01	0.01	0.00	0.01
O2	0.02	0.00	0.01	0.00
O3	0.02	0.00	0.01	0.02

Table S3. Intermolecular coordination numbers (CN) at the distances given in the table as extracted from the MD simulations of *cis* GPG. It can be seen that the coordination numbers are small, i. e. there is no appreciable aggregation of different molecules in the simulation.

	Hn1 (2.3 Å)	Hn3 (2.8 Å)	Hn4 (2.5 Å)	Hn4′ (2.5 Å)
O1	0.01	0.01	0.01	0.01
O2	0.01	0.02	0.01	0.02
O3	0.01	0.02	0.01	0.02

Table S4. Intermolecular coordination numbers (CN) at the distances given in the table as extracted from the MD simulations of *trans* GPG. It can be seen that the coordination numbers are small, i. e. there is no appreciable aggregation of different molecules in the simulation.

	Hn1 (2.3 Å)	Hn3 (2.8 Å)	Hn4 (2.5 Å)	Hn4′ (2.5 Å)
O1	0.01	0.02	0.01	0.02
O2	0.01	0.03	0.01	0.02
O3	0.02	0.03	0.02	0.02

Finally, the pressure was allowed to equilibrate within the system by carrying out a simulation using the NPT ensemble at a temperature of 300 K and a pressure of 1 atm, which was 2 ns in duration. Finally, the production simulations were conducted using the NPT ensemble, where the temperature was 300 K and the pressure was 1 atm, which were run for 40 ns using a 2 fs timestep. The configuration of the system was saved in steps of 10 ps for the analysis. In all simulations, the temperature is controlled using the Nose-Hoover thermostat[12,13] and the pressure is controlled using the Martyna-Tuckerman-Tobias-Klein (MTTK) barostat.[14] The van der Waals interactions were cutoff at 14 Å, while the particle mesh Ewald (PME) algorithm[15,16] was used to compute the long-range Coulomb interactions.

The CHARMM forcefield that was used to model the GPG molecules in the MD simulations utilized the CMAP term, which is a grid-based correction for the ϕ-ψ-angular dependence of the energy. This correction has been shown to provide significant improvements in the residue-location specific distribution of dihedral angles in protein in solution simulations.[17] In following with the standard implementation of the CMAP term, the correction is not applied to either of the two terminal Gly residues.

Figures of molecular configurations were produced using Aten,[18] Avogadro,[19] POV-ray,[20] VMD[21] and Inkscape.[22] Scattering functions and radial distribution functions (from both, MD and EPSR[9]) were plotted using a toolchain based on Python,[23] IPython,[24] matplotlib[25] and scipy.[26] For plots of spatial density functions,[27] Mayavi[28] was used in addition.

Details of the NMR experiments

NMR spectra were acquired using home-built 500 MHz and 750 MHz spectrometers at the University of Oxford which are controlled with GE/Omega software and equipped with a home-built triple-resonance pulsed-field-gradient probehead. For all the experiments, the sample temperature was set to 20°C. The full ^1H spectrum for GPG in H_2O is shown in Fig. S4, the concentration of the GPG sample was the same as in the neutron measurements. From this figure the different *cis* versus *trans* peaks can be seen. The spectra from two more dilute solutions of GPG in water were also obtained to ensure that the *cis*/*trans* ratio was not concentration dependent.

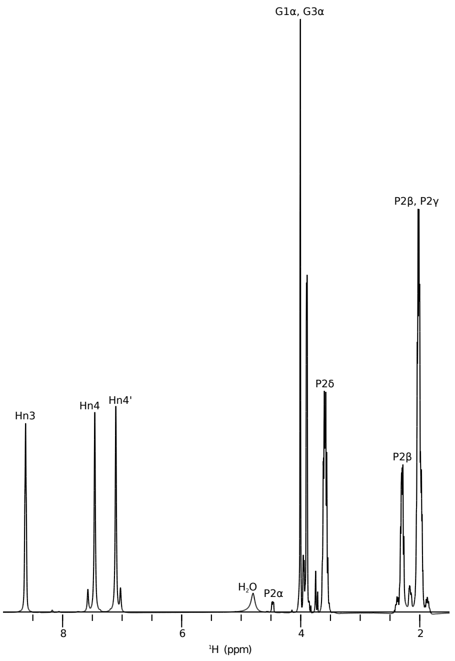

Figure S4. 1D ^1H NMR spectrum for GPG in H_2O. The names are given in Fig. S5.

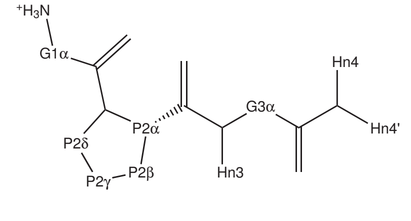

Figure S5. Naming convention used for the NMR spectra shown in Figs. S4 and S6. The NH_3^+ group is also named for clarity although it is not visible in the NMR spectra.

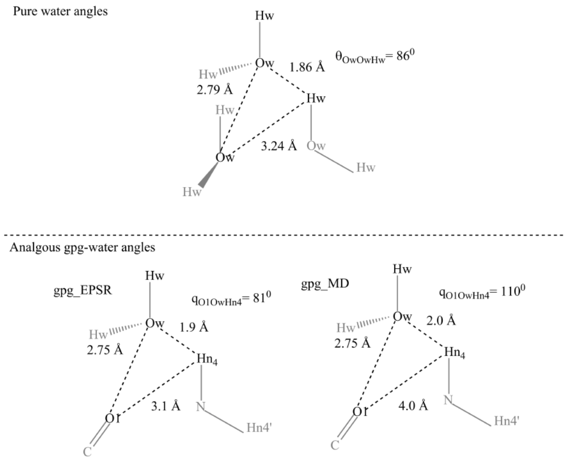

Figure S7. Average O1$\cdots$Ow$\cdots$Hn4 angles for GPG in solution from MD simulations and EPSR fits to the neutron diffraction data compared with the analgous angle in pure water.[33]

The NMR spectra were processed and visualized using NMRPipe, NMRDraw, and CcpNmr Analysis,[29,30] the figures were produced with Inkscape.[22]

The HSQC spectrum of GPG in D_2O at a concentration of 0.09M is shown in Fig. S6, the highlighted portion of this spectrum is shown in the main text. Fig. S6 shows the typical peaks for *cis* and *trans* X-Pro peptide bonds. The peaks are labelled according to Fig. S5 and are denoted with c and t for the *cis* and *trans* conformer, respectively. Concentrated GPG in solution gave similar results (not shown). The chemical shift differences between the *cis* and the *trans* conformation of GPG are in agreement with literature values.[31,32]

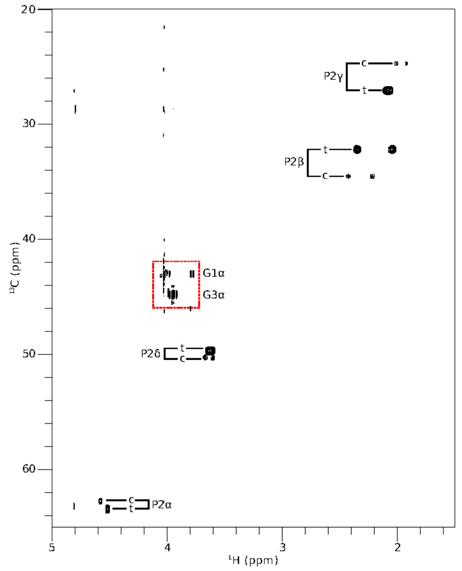

Figure S6. ^1H–^{13}C HSQC NMR spectrum of GPG. The atom names are given in Fig. S5; c and t denote peaks originating from the *cis* and *trans* conformer, respectively. The red dotted line around the G1α and G3α resonances highlights the part of the spectrum that is shown in the main part of the manuscript.

Water-mediating angle calculation

Figure S7 shows the bond angle calculation for the O1$\cdots$Ow$\cdots$Hn4 angles for both MD and EPSR simulations compared with analgous calculations for the structure of pure water. The distances for water were taken from distances reported in Ref. [33].[33]

References

[1] V. F. Sears, *Neutron News* **1992**, *3*, 26–37.

[2] A. K. Soper, GudrunN and GudrunX, **2012**.

[3] A. K. Soper, W. S. Howells, A. C. Hannon, ATLAS – Analysis of Time-of-Flight Diffraction Data from Liquid and Amorphous Samples, Report RAL-89-046, Rutherford Appleton Laboratory, **1989**.

[4] A. K. Soper, *Molecular Simulation* **2012**, *38*, 1171–1185.

[5] A. D. MacKerell, Jr., *Journal of Computational Chemistry* **2004**, *25*, 1584–1604.

[6] A. D. MacKerell, Jr., D. Bashford, M. Bellott, J. R. L. Dunbrack, J. D. Evanseck, M. J. Field, S. Fischer, J. Gao, H. Guo, S. Ha, D. Joseph-McCarthy, L. Kuchnir, K. Kuczera, F. T. K. Lau, C. Mattos, S. Michnick, T. Ngo, D. T. Nguyen, B. Prodhom, I. W. E. Reiher, B. Roux, M. Schlenkrich, J. C. Smith, R. Stote, J. Straub, M. Watanabe, J. Wiorkiewicz-Kuczera, D. Yin, M. Karplus, *J. Phys. Chem. B* **1998**, *102*, 3586–3616.

[7] W. L. Jorgensen, J. Chandrasekhar, J. D. Madura, R. W. Impey, M. L. Klein, *The Journal of Chemical Physics* **1983**, *79*, 926.

[8] W. I. Reiher, PhD thesis, Harvard University, **1985**.

[9] A. K. Soper, *Physical Review B* **2005**, *72*, 104204.

[10] S. E. Rogers, A. E. Terry, M. J. Lawrence, J. Eastoe, J. T. Cabral, A. Chan, *Materials Today* **2009**, *12*, 92–99.

[11] B. Hess, C. Kutzner, D. van der Spoel, E. Lindahl, *Journal of Chemical Theory and Computation* **2008**, *4*, 435–447.

[12] S. Nosé, *Molecular Physics* **1984**, *52*, 255–268.

[13] W. Hoover, *Physical Review A* **1985**, *31*, 1695–1697.

[14] G. J. Martyna, M. E. Tuckerman, D. J. Tobias, M. L. Klein, *Molecular Physics* **1996**, *87*, 1117–1157.

[15] T. Darden, D. York, L. Pedersen, *The Journal of Chemical Physics* **1993**, *98*, 10089.

[16] U. Essmann, L. Perera, M. L. Berkowitz, T. Darden, H. Lee, L. G. Pedersen, *The Journal of Chemical Physics* **1995**, *103*, 8577.

[17] M. Buck, S. Bouguet-Bonnet, R. W. Pastor, A. D. MacKerell, Jr., *Biophysical Journal* **2006**, *90*, L36–L38.

[18] T. G. A. Youngs, *Journal of Computational Chemistry* **2009**, NA–NA.

[19] M. D. Hanwell, D. E. Curtis, D. C. Lonie, T. Vandermeersch, E. Zurek, G. R. Hutchison, *Journal of Cheminformatics* **2012**, *4*, 17.

[20] POV-ray, http://www.povray.org/, `http://www.povray.org/`.

[21] W. Humphrey, A. Dalke, K. Schulten, *Journal of Molecular Graphics* **1996**, *14*, 33–38.

[22] Inkscape, http://www.inkscape.org/, `http://www.inkscape.org/`.

[23] G. van Rossum et al., Python, http://www.python.org/, `http://www.python.org/`.

[24] F. Perez, B. E. Granger, *Computing in Science & Engineering* **2007**, *9*, 21–29.

[25] J. D. Hunter, *Computing in Science & Engineering* **2007**, *9*, 90–95.

[26] T. E. Oliphant, *Computing in Science & Engineering* **2007**, *9*, 10–20.

[27] A. K. Soper, *The Journal of Chemical Physics* **1994**, *101*, 6888.

[28] P. Ramachandran, G. Varoquaux, *Computing in Science & Engineering* **2011**, *13*, 40–51.

[29] F. Delaglio, S. Grzesiek, G. W. Vuister, G. Zhu, J. Pfeifer, A. Bax, *Journal of Biomolecular NMR* **1995**, *6*, 277–293.

[30] W. F. Vranken, W. Boucher, T. J. Stevens, R. H. Fogh, A. Pajon, M. Llinas, E. L. Ulrich, J. L. Markley, J. Ionides, E. D. Laue, *Proteins: Structure Function and Bioinformatics* **2005**, *59*, 687–696.

[31] M. J. O. Anteunis, F. A. M. Borremans, J. M. Stewart, R. E. London, *Journal of the American Chemical Society* **1981**, *103*, 2187–2191.

[32] Y.-C. Lee, P. L. Jackson, M. J. Jablonsky, D. D. Muccio, R. R. Pfister, J. L. Haddox, C. I. Sommers, G. M. Anantharamaiah, M. Chaddha, *Biopolymers* **2001**, *58*, 548–561.

[33] A. K. Soper, *ISRN Physical Chemistry:2013* **2013**, Article ID 279463.
